# Supplementary material for: A rubric for assessing conformance to the Ten Rules for credible practice of modeling and simulation in healthcare
Source: PLoS One. 2025 Jun 25;20(6):e0313711. doi: 10.1371/journal.pone.0313711 (PMC12192136; doi:10.1371/journal.pone.0313711)
Supplement: S2 File — (DOCX) [file pone.0313711.s002.docx]

Commentary on the review of CHIME v1.1.2

*as performed by Ahmet Erdemir during April 5-6, 2019*

*This document by Ahmet Erdemir,* [*erdemira@ccf.org*](mailto:erdemira@ccf.org)*, April 13, 2020.*

The overall intention of the review was to understand the credibility potential of the modeling and simulation lifecycle of CHIME v1.1.2. The review leveraged the recommendations of Ten Simple Rules for Credible Practice of Modeling and Simulation in Healthcare, which indicated key activities that can enhance the credibility potential of a modeling and simulation workflow and subsequently, resulting models and predictions. The review also relied on a rubric to evaluate conformance level of the practitioners to each of the Ten Simple Rule. An important distinction of the rubric was that conformance level was an indicator of the accessibility and outreach potential of the followed practice by other modeling & simulation practitioners and/or application domain experts. The perceived conformance level to the rules by the reviewer, in light of the rubric was:

**Comprehensive** RULE 1 – Define context clearly

**Adequate** RULE 2 – Use contextually appropriate data

**Insufficient** RULE 3 – Evaluate within context

**Adequate** RULE 4 – List limitations explicitly

**Extensive** RULE 5 – Use version control

**Extensive** RULE 6 – Document adequately

**Comprehensive** RULE 7 – Disseminate broadly

**Insufficient** RULE 8 – Get independent reviews

**Partial** RULE 9 – Test competing implementations

**Adequate** RULE 10 – Conform to standards

Clear delivery of the targeted context of use for the model is arguably the most important activity to render the practice as credible. Intended use of the tool indicates potential consequences of decision making based on the modeling and simulation activity and the desired conformance level to other rules will need to match these. The modeling and simulation lifecycle of CHIME has been captured through a web site, which provides the entry to the tool and all related activities. The goals of potential uses of the model are clearly described albeit some of this information are in deeper layers of this presentation (such as target end-users and their intended utilization). Given the primary context of use for the tool, i.e. hospital capacity planning, the stakes are high as it will likely inform hospital management decisions and regional policy making. Portrayal of data curation, analysis, and its use for modeling and simulation, particularly to set default model parameters, is at a level accessible to the modeling and simulation practitioners in the application domain. Data are traceable but in some cases, not in a clear fashion for an outsider, i.e. transformation of some of the raw data from unpublished literature to model inputs is not elaborated as much. In addition, data to support evaluation of modeling and simulation within its context of use is largely unreported. Yet, this is understandable as such an evaluation has not been performed. Rather, a third party review was relied on. Conformance to the rule related to evaluation of model and simulation results can be enhanced by leveraging prior data and performing sensitivity analysis. However, these activities seemed to be left to the end-user. Disclaimers on model reuse have been provided but their indications are probably interpreted at best by the modeling and simulation experts in this specific application domain. Practices related to version control, documentation, and dissemination are in-depth and accessible to modelers that are not in the application domain and in a few cases to domain experts that are not modelers. Additional guidance to navigate such comprehensive information (versioning strategies, user and developer documents, and shared modeling and simulation products) will likely enhance outreach capacity. The attempt to get independent reviews is commendable. Nonetheless, the outcomes of such reviews have not been provided and therefore, reduces the perception of the practice’s credibility. Simulations to compare against another modeling activity are welcome but are confined to certain aspects of modeling. Comparisons against additional competing modeling implementations in relevance to the context of use (predictions related to regional hospital capacity) may improve conformance level of this activity. Utilization of a common modeling form in epidemiology and adoption of broadly known free and open source approach indicate the developer’s intentions to conform to standards but perhaps the use of standards in this specific domain is not well established. Overall, recommended enhancements to the modeling and simulation practice and the delivery of its products to prospective end-users will likely increase their perceived level of credibility. Due to the high stakes of the tool’s intended use, such enhancements have critical importance.

This exercise indicated some limitations related to the Ten Simple Rules and the design of the rubric. Ten Simple Rules provide short and long descriptors of the rules, which are supported by elaboration in a manuscript text. Due to the ambiguity of natural language, short descriptors, while mining a lot of information, may lead to misunderstandings. It is recommended that when Ten Simple Rules are used for assessment of credibility, their users pay attention to the specific details of these rules. An important consideration is to also acknowledge that Ten Simple Rules refer to the credibility of whole modeling and simulation practice not just the model itself. In regard to the rubric, it is advised to use numerical scaling for conformity levels rather than adjectives such as “comprehensive”, “adequate”. Commonly appreciated meanings of these terms may cloud their intended meaning for the rubric. For some rules, a detailed description of how the practice can conform to the rule indicates multiple conditions. This may result in grading that is not fitting to any level due to conflicting performances for different conditions. This exercise also points out to the need for rubrics to help decision makers evaluate such credibility reports while balancing influence vs consequence, risk vs benefit, and in the context of this modeling activity, likelihood of a prediction vs availability of resources to act upon that prediction.
