# Supplementary material for: A rubric for assessing conformance to the Ten Rules for credible practice of modeling and simulation in healthcare
Source: PLoS One. 2025 Jun 25;20(6):e0313711. doi: 10.1371/journal.pone.0313711 (PMC12192136; doi:10.1371/journal.pone.0313711)
Supplement: S3 File — (DOCX) [file pone.0313711.s003.docx]

**Summary of Credible Practice Review**

**Model Name:** CHIME

**Model version:** v1.1.2 (2020-04-01)

**Reviewer:** Lealem Mulugeta

**Reviewer number:** 2

**Original review submission date:** April 8, 2020

**Review summary submission date:** April 16, 2020

This is an overview of the rigor of credible practice of modeling & simulation employed by the investigators of the COVID-19 Hospital Impact Model for Epidemics (CHIME) model to develop and communicate guidance on the contextually appropriate use of CHIME. This credible practice review should not be interpreted as a credibility or accreditation of the CHIME model for its contextual application.

The review was performed using the Ten Simple Rules of Credible Practice of Modeling & Simulation in Healthcare. The rigor of conformance to the ten simple rules was evaluated using a gradient rubric for outreach potential to M&S practitioners and application domain experts. To complement this summary report, an overall conformance to credible practice has been assessed (Table 1). This final assessment score assumes each of the importance of the ten rules are weighted equally.

**Table 1:** Summary of Conformance Level to the Ten Simple Rules of Credible Practice of Modeling & Simulation in Healthcare

| **Ten Simple Rules of Credible Practice** | **Conformance (level)** |
| --- | --- |
| **Rule 1:** Define context clearly | Adequate outreach (3) |
| **Rule 2:** Use contextually appropriate data | Partial outreach (2) |
| **Rule 3:** Evaluate within context | Insufficient outreach (1) |
| **Rule 4:** List limitations explicitly | Partial outreach (2) |
| **Rule 5:** Use version control | Extensive outreach (4) |
| **Rule 6:** Document appropriately | Extensive (4) |
| **Rule 7:** Disseminate broadly | Comprehensive (5) |
| **Rule 8:** Get independent review | Partial (2) |
| **Rule 9:** Test competing implementations | Partial (2) |
| **Rule 10:** Conform to standards | Comprehensive (5) |
| ***Overall conformance to TSR:*** | ***Adequate (3)*** |

**Strengths:**

The investigators of the CHIME did extremely well by providing the accessibility of the model, all versions code, open source/access methods, standardized coding practices, and necessary documentation needed to use and develop the model. Furthermore, they continue to update the model regularly in response to user feedback and rapidly evolving conditions (v1.1.3 released on April 8^th^). This level transparency and engagement with the stakeholder community can be an asset for advancing the state of knowledge and robustness of how CHIME is developed and implemented. For these reasons, rigor of conformance to Rules 5, 6, 7 and 10 was deemed to be extensive (4) or comprehensive (5).

**Weaknesses:**

The level of information available on the quality of data used to contextually develop and evaluate CHIME is quite lacking. If this information is available, it should be documented more clearly and made easily accessible.

Tangible information is not available contextual evaluation of the model. Specifically, this reviewer was not able to find any details on verification, validation, uncertainty quantification and sensitivity analysis of the model. Moreover, the developer’s guide clearly states the CHIME model lacks a validation routine, and the investigators are seeking help from the developer community. However, the documentation makes no distinction between verification and validation. Validation and testing seem to be interchangeably to mean both verification and validation.

The primary evaluation sources for CHIME seems to be a comparison the Imperial College COVID-19 Publication, and independent review by several epidemiologists. However, comparison with the Imperial College published results assumes the Imperial College model is a gold standard. Also, credible practice for independent review of models should require the investigators to publish the details of what the reviewers did to evaluate the model, as well as their conclusions about the model’s capability to be used within the stated context. So it is this reviewer’s opinion the information provided regarding the subject matter expert reviews of the CHIME model is too limited to understand the value of their reviews.

On the landing page of the CHIME model, the investigators acknowledge the high degree of uncertainty regarding the details of how COVID-19 propagates and the efficacy of current mitigation measures. Therefore, they urge extreme caution in the use of the model for long-term projections. However, the investigators do not define what “long-term” means within the context of their modeling approach and the model context of use. Such a disclaimer should warrant sensitivity analysis of the key parameters, error propagation across parameters, and uncertainty quantification across prediction timeframe.

The context of use of this model was adequately clear regarding the target audience (i.e. hospitals and health officials) in relation to COVID-19. But more detail is necessary to help the intended users to apply the model appropriately. For example, how far does one interpret the statement “The CHIME APP is designed to assist hospitals and public health officials understand hospital capacity needs…”? Does capacity also include physicians, nurses, medical technicians, laboratories, bedding materials, and more? This becomes even more ambiguous when the end-user is expected to take into account the caution against using CHIME for “long-term” projections. If the intention is to help hospitals and health officials make decisions about capacity planning, it is important to also specify what aspects of capacity planning it is useful for, the extent it can be applied confidently, as well as how it should NOT be applied.

Clear and accurate definition of the context of use is paramount because it sets the tone on the level of rigor one must comply with the remainder of the rules. It is possible the reason why there were significant deficiencies in the data and evaluation methods was due to the insufficient clarity on the model context of use.

| Project Title: | COVID-19 Hospital Impact Model for Epidemics (CHIME) |
| --- | --- |
| Investigators | Predictive Healthcare team at Penn Medicine  <http://predictivehealthcare.pennmedicine.org/team/> |
| Contact email | pennsignals@uphs.upenn.edu |

| **Rule 1: Context of Use** |
| --- |
| **IMAG Wiki form:**  Aid in Clinical decision making  *“to assist hospitals and public health officials understand hospital capacity needs”*  **Per TSR manuscript**  **Domain of Use:** Clinical Care and Public Healthcare  **Use Capacity:** Therapeutics Implementation  *“CHIME enables capacity planning by providing estimates of total daily (i.e. new) and running totals of (i.e. census) inpatient hospitalizations, ICU admissions, and patients requiring ventilation.”* |
| **Conformance Level (Strength of Influence per TSR manuscript)**  Adequate:  *Summary is understandable by M&S practitioners and application-domain experts;*  *Detailed explanation is understandable by M&S practitioners familiar with the application-domain;* |
| **Primary Goal of the model/tool/database**  To assist hospitals and public health officials understand hospital capacity needs as they relate to the COVID-19 pandemic. CHIME enables capacity planning by providing estimates of total daily (i.e. new) and running totals of (i.e. census) inpatient hospitalizations, ICU admissions, and patients requiring ventilation. These estimates are generated using a SIR (Susceptible, Infected, Recovered) model, a standard epidemiological modeling technique. |
| **Biological domain:** Population (basing this on SIR models falling under Population dynamics)  **Structures of interest in the model:** Typical of SIR Model  **Spatial scales included in the model:** Patient level  **Time scales included in the model:** Days |
| **Other uses for the model (Optional)**  None stated. However, it may be possible to augment the code to assess implications managing hospital capacity in the event essential medical staff are also infected or succumb to COVID-19. |
| **Additional comments about the model’s context (optional)**  None |
|  |

| **Rule 2: Use contextually appropriate data** |
| --- |
| **Data for building and validation the model**  Clinical data was used to develop the model  *The estimated number of currently infected individuals is 20128. This is based on current inputs for Hospitalizations (69), Hospitalization rate (2%), Region size (3600000), and Hospital market share (15%).*  **Data used for validating the model**  The primary validation data source seems to be a comparison of the CHIME model against Imperial College COVID-19 Publication. In order to establish high confidence in the CHIME model based on this comparison would be predicated on the acceptance that the Imperial College model is the gold standard.  Furthermore, it is not clear if and what additional data was collected and used by the CHIME team to validate the model. The investigators only indicate the model has been reviewed by several epidemiologists. The number of reviewers, form and rigor of the evaluations is not stated. |
| **Conformance Level per TSR Rubric**  Because the documentation does not discuss the data source and origin sufficiently, the conformance of the data used is “Partial”  *Part of the data used in M&S development and/or operation is traceable to its original source; M&S practitioners familiar with the application-domain can understand some aspects of how the data was used;* |

| **Ruel 3: Evaluate within context** | | | | |
| --- | --- | --- | --- | --- |
|  | **Who does it** | **When does it happen** | **How is it done** | **Conformance level** |
| Verification | Unclear | Unclear | Unclear | Insufficient |
| Validation | CHIME team & SMEs | Unclear | Mostly unclear, but the prediction capacity of the model was compared against the Imperial Collage COVID-19 model. See notes in Rule #2 | Partial |
| Uncertainty Quantification | Unclear | Unclear | Unclear | Insufficient |
| Sensitivity analysis | Unclear | Unclear | Unclear | Insufficient |
| The documentation in the “Manual Validation” portion of the contributor manual provides “Manual Testing Procedures”. In reading the procedures, there seems to be a lack of distinction between verification, validation, uncertainty quantification. In fact the procedures outlined do not use any of these terms, even though the table of contents uses the term validation.  Furthermore, at the very bottom of the “Contributing: Application Development” page, the developer guide states “No validation routine is available yet. If you have thoughts on how to add one, please contribute!”  However, the CHIME team has not provided clear terminology in their glossary of terms for what validation means or the different phases of model testing.  **OVERALL CONFORMANCE LEVEL:** Insufficient | | | | |

| **Rule 4: List limitations explicitly** |
| --- |
| The investigators clearly state the unknowns about the disease and what it means for long-term predictions using the model.  **Notice**: There is a high degree of uncertainty about the details of COVID-19 infection, transmission, and the effectiveness of social distancing measures. Long-term projections made using this simplified model of outbreak progression should be treated with extreme caution.  However, the investigators do not state what “long-term” is defined as. In other words, the validation time range domain is not clearly stated. Nor do they state the limitations for population size and other parameters of the model. Given the novelty of the disease and the lack of clarity about the quality of the data used, it is likely there are other areas of limitations the end-users need to know about. |
| **Who needs to know about it:** Medical practitioners, hospitals and policy makers |
| **How is the disclaimer shared with that audience**: The disclaimer is stated at the very beginning of the CHIME browser interface. |
| **Conformance level:** Partial  *Some of the limitations understandable by M&S practitioners that are familiar with the application-domain;* |

| **Rule 5: Use Version Control** |
| --- |
| All version of code with release notes are tracked and shared using Github. Sufficient documentation and manuals are available to allow developers contribute or adapt the model to their needs. However, one must be an M&S practitioner to fully understand the version history and contributions. |
| **Conformance level:** Extensive  *Extensive version control history available to an independent third party; Version history and contributions are understandable by M&S practitioners that may not be familiar with the application-domain;* |

| **Rule 6: Document adequately** | | | |
| --- | --- | --- | --- |
|  | **Conformance** | **Rubric description** | **Notes** |
| **Code commented?** | N/A |  | I was not able to review the code, so difficult to say. |
| **Scope and intended use described** | Adequate/Partial | Partial/Limited documentation regarding limitations and VVUQ.  Limited user guide, READMEs, or manuals; Understandable by M&S practitioners that are familiar with the application-domain; | This can be updated to extensive or comprehensive once the limitations and VVUQ concerns are addressed. |
| **User’s guide?** | Comprehensive | Detailed user guide, READMEs, or manuals; Understandable by application-domain experts that may not be M&S practitioners; | There is sufficient documentation to allow users to apply the model. There is also a slack workspace. |
| **Developers guide?** | Extensive | Extensive mark-up of the model and simulation code; Detailed developer guide, READMEs, or manuals; Understandable by M&S practitioners that are not familiar with the application-domain; | There are portions of the developer guide missing information. There is also a slack workspace. |

| **Rule 7: Disseminated Broadly** |
| --- |
| **Conformance:** Comprehensive/Extensive  They are comprehensive when it comes to sharing all aspects of the model. They are lacking however, elements of the data and evaluation mentioned in Rules 2-4. |

| **Rule 8: Get independent reviews** |
| --- |
| The CHIME team indicate the model has been reviewed by several epidemiologists. The number of reviewers, form and rigor of the evaluations is not stated. Only one epidemiologist is listed by name in this statement.  **Reviewer:** Michael Z. Levy, PhD - Perelman School of Medicine, University of Pennsylvania  **Period of review:** Unknown  **Method of review:** Unknown  **Conformance level:** Partial  *M&S activity partly reviewed by third party M&S practitioners that are familiar with the application-domain;* |

| **Rule 9: Test competing implementation** |
| --- |
| The model was only compared with the Imperial College COVID-19 publication. It is not clear if there were any model refinement or improvements made as an outcome of this comparison.  **Conformance level:** Partial  *Two contrasting implementations are partly tested and compared; Implementations partly understandable by M&S practitioners that are familiar with the application-domain;* |

| **Rule 10: Conform to standards** |
| --- |
| **Conformance level**: Comprehensive  *Follows best practices that are in use by application-domain experts that may not be M&S practitioners; Use of model formats and/or simulation tools popular in or relevant to an organ/disease specific research community;* |
