# Supplementary material for: A rubric for assessing conformance to the Ten Rules for credible practice of modeling and simulation in healthcare
Source: PLoS One. 2025 Jun 25;20(6):e0313711. doi: 10.1371/journal.pone.0313711 (PMC12192136; doi:10.1371/journal.pone.0313711)
Supplement: Attachment — Submitted filename: Response-to-reviewers-20March2025.docx [file pone.0313711.s008.docx]

**Response to Reviewers:**

**Reviewer #1:**

**Comment 1.** Overall, I really think you are onto something here. I can see hints of how something like what you are talking about can be very useful and how it may actually side step some of the issues that others have fallen into when trying to provide similar information about models and simulations. For example, what you are talking about here has been tried by the national labs with PCMM. Only it didn't work. I think they even revised it 4 or 6 times, and they got it to a good point, but it never could provide that "single number" that everyone wants.

However, I ultimately got very confused about the purpose of the rubric. Is it a measure of the credibility of a model/simulation or is it a measure of the availability and "understandability" of the information needed to determine the credibility of a model/simulation?

Consider two different simulations. For the first simulation, assume I have followed Ten Rules to such an extent than anyone who saw my work would immediately agree that I clearly followed all Ten Rules. However, assume that for this simulation I am not providing ANY information such that someone else could independently determine that I followed any of the rules. Would the rubric rate this scenario high or low?

Conversely, consider a situation in which I followed none of the rules. However, I have provided the information such that everyone can quickly determine that I have followed none of the Ten Rules. Would the rubric rate this scenario high or low?

I feel like "large changes" are needed to this paper, not because a lot of wording needs to change. Overall, the paper is well written. However, I feel like what the paper is saying needs to be made much clearer. I think this can be done with a few wording changes, but I think it is extremely important as those wording changes define the rubric, and right now I do not understand the rubric at all.

Just to re-iterate, even though I say "major revision", I don't expect to see that many wording changes in the paper, but there needs to be a further definition of just what the rubric is, and since that is the heart of the paper, that is a "major change" to me.

**Response:** We thank the reviewer for their comments and appreciate the support. We would like to clarify that purpose of the rubric is not to measure the accuracy of a model/simulation but rather to evaluate the conformance level of an M&S study to the Ten Rules for credible practice based on outreach potential.

We appreciate the two scenarios you describe for their potential in clarifying the use of the rubric to assess credible practice of a ***modeling study*** and not in validating or verifying a specific model, i.e., the Ten Rules and the associated rubric apply to the practice of M&S, not to a model.

When one considers the two different scenarios you posed above, both would result in a rubric rating of 0. In the first scenario, you explain that you have followed the Ten Rules extensively but have not provided any information for independent determination of the extent to your conformance. This would result in a rubric score of 0 because the purpose of the rubric is to assess conformance to the Ten Rules of credible practice as a function of outreach to various stakeholders. If none of the stakeholders are able to determine the extent to which one has followed the rules, then the conformance of the ***modeling*** ***study*** (not the model) to the Ten Rules for credible practice cannot be determined and hence the score would equal to 0.

In the second scenario, where one has not followed any of the rules but has provided information such that others can also determine that one has not followed any of the rules. In this situation, all stakeholders assessing the conformance of that modeling study to the Ten Rules for credible practice would then agree that the study investigators have not conformed to any of the Ten Rules and therefore the ***study*** should receive a score of 0.

**Action taken:** We have included additional text throughout the revised version of the manuscript to clarify and explain the purpose of the rubric more explicitly. We included the explanation above two scenarios to clarify the use of the Ten Rules and the assessment rubric.

**Comment 2.** This paper was very well written. I have a few comments here and there, but my main comment is that I didn’t understand the main purpose for the rubric. Is the rubric supposed to communicate information about the Ten Rules or be used to evaluate if the Ten Rules have been followed? I have added additional comments in the paper, but I wanted to raise this issue here since the abstract makes it seem like the rubric is only for communication and not evaluation.

*In reference to “This rubric considers the ability of the M&S to facilitate outreach of the results to a wide range of stakeholders from context-specific M&S practitioners to policymakers.” in the abstract.*

**Response:** We point the reviewer to our response to Comment 1. The rubric is to be utilized in conjunction with the Ten Rules to assess M&S conformance to the credible practice of modeling based on outreach capability to a wide range of stakeholders. It is not for evaluation of the model *per se* but is for assessment of the underlying process/practice.

**Action taken:** We adjusted the sentence in the abstract to read as “This rubric considers the ability of an M&S study to communicate how well the study conforms to the Ten Rules for credible practice and facilitate outreach to a wide range of stakeholders from context-specific M&S practitioners to policymakers.”

**Comment 3.** On a first read of this phrase, I felt like phrase was awkward "assessing the conformance of an M&S activity to the Ten Rules".

After re-reading it a few times, I think it may be correct, but it caused me to stumble when I reading the abstract. It just seems strangely worded. I don't really every think of "assessing the conformance of an activity to some set of criteria".

I realize this is also the title of the paper, and if your community uses the phrase “assessing conformance to X” in other documents and your audience is familiar with that phrasing, then I would keep the wording the same and the title the same. However, if that is not a common phrase in your community, I suggest changing both as I think they don’t easily express the idea they are trying to convey.

*In reference to “assessing the conformance of an M&S activity to the TR” in the abstract.*

**Response:** We thank the reviewer for their comment. It is common to use the phrase “assessing conformance to X” so we have chosen to keep this verbiage throughout the manuscript.

**Comment 3.** I like the fact that you call it out as an ordinal scale. However, if the scale is truly ordinal (which I very much believe it is), then combining the numbers is to an average is not mathematically supported and has no real inherent meaning. For example, lets say we are only focused on two of the ten rules and we average the results from those two. If I told you the average was "2", you would not know if the scores from those two rules was (2,2) or (4,0) or (0,4), or (3,1), or (1,3). If there is no difference between those five sets of scores (i.e., getting any one of those scores is identical to getting another), then averaging makes sense. However, if you believe there is a large difference in getting those scores, then averaging makes no sense since it can not distinguish between those cases.

**Response:** We appreciate the reviewer’s concern for the numerical scoring method that is to be utilized with the rubric. We would like to clarify that we aim to utilize the scoring metric as an indicator for assessing the extreme cases of M&S conformance. We agree that the proposed numerical score has its limitations, i.e., a moderate score does not directly inform the intended audience of the individual conformance levels for each of the TR. However, at the high and low extremities, one can readily determine exceptional vs. poor M&S conformance to credible modeling practices using the proposed summative scoring method. That said, we agree with the reviewer that a more granular numerical scoring approach can help develop a deeper understanding of the M&S study’s conformance level when the summative total numerical score across the TR is in the moderate range. Therefore, in the Discussion section, we have proposed the use of a categorized rubric with combined numerical scores for M&S Development (including Rules 1, 3, 5, and 10), M&S Application (Rules 2 and 4), and M&S Supporting Evidence (Rules 6, 7, 8, and 9) that strikes a balance. Another alternative is to develop visual representations, for example using radar/spider plots, that can illustrate the multiple levels of conformance across the TR without combining into a single numerical score. While such a representation would show the granular information for every rule, it can become complicated to parse the information for an overall summative interpretation of the TR.

**Action taken:** We have included additional text in the revised version of the manuscript to further explain the intended purpose of the ordinal scale associated with the TR rubric.

**Comment 4.** To go back to my main confusion, the above statement talks about outreach of the results, but this statement talks about assessment of the results. I can't tell what you intended the rubric for.

*In reference to “providing a uniform approach for comparing assessments across different reviewers and different models” in the abstract.*

**Response:** We kindly refer the reviewer to our responses to the previous comments, where we explain our intended use of the rubric.

**Action taken:** We have adjusted the abstract text to clarify that the rubric is to be used to evaluate the ability of an M&S study to facilitate outreach to a range of stakeholders. The ordinal scale, which is applicable to each rule of the Ten Rules, was developed to provide a uniform approach for comparing assessments across reviewers that are assessing the conformance of a given M&S study/practice/process as well as for comparing across different modeling studies.

**Comment 5.** Here, you seem to make it clear that the rubric is for assessing the model. I do not understand how the rubric can be used for that. Its possible that I don't understand the rubric, but to me it does not provide an assessment of the model, only an assessment of if the information concerning the VVUQ of a model is or can be widely understood.

*In reference to “We used the rubric to evaluate the conformance of two computational modeling activities:” in the abstract.*

**Response:** We kindly refer the reviewer to our responses to the previous comments, where we explain our intended use of the rubric. In this sentence, we state our intended use of the rubric in evaluating the conformance of two computational modeling *activities* (and not the models) to the Ten Rules.

**Comment 6.** I think the rubric is a good to, but I don't see it assessing the model.

*In reference to “Our findings demonstrate that the TR rubric represents a systematic tool for assessing the conformance of an M&S activity to codified good practices and enhances the value of the TR for supporting real-world decision-making.” in the abstract.*

**Response:** We kindly refer the reviewer to our responses to the previous comments, where we explain our intended use of the rubric. In this sentence referenced, we do not state that the TR rubric is used to assess the model but rather to assess the conformance of an M&S activity.

**Comment 7.** I can understand referencing the NASA document, although there is a later version than what you have referenced. I am really surprised you don't reference V&V 40, since that is the main standard in this area. The National Research Council document isn't a standard, its a report. Also, I don't think its a bad thing to reference the paper by the FDA folks, but why not reference the document they just put out on "Assessing the Credibility of Computational Modeling and Simulation in Medical Device Submissions"?

*In reference to citations to citations (3–5) on Line 58.*

**Response:** We thank the reviewer for pointing this out and we apologize for our oversight.

**Action taken:** We have updated the references to include the V&V40 and the FDA paper in the revised version of the manuscript.

**Comment 8.** It seems weird to call a paper a standard. I am not saying its wrong, it may be appropriate. I just wanted to highlight it so you could make sure this is what you intended to do....

oh, I see.

You are not calling this document a standard, but you are saying that this document reviewed standards.

It does seem a bit odd to talk about "multiple standards" and then not provide a direct reference to them, but a reference to a document which reviews them.... especially when you talk about the same thing in the next paragraph.

I am not sure this sentence is needed here, but I leave it up to you.

*In reference to “Similarly, multiple standards exist for systems biology applications aimed at addressing conceptual information, nomenclature, data formats, and representations of biochemical systems, and are intended to improve the communication and sharing of M&S components (6).” beginning on Line 61.*

**Response:** We thank the reviewer for their comments and regret the confusion caused.

**Action taken:** We have updated this sentence for clarification to read “Similarly, multiple standards exist for systems biology applications, which have been reviewed in Tatka et al. 2023 [(6)](https://sciwheel.com/work/citation?ids=15195376&pre=&suf=&sa=0), that aim to address conceptual information, nomenclature, data formats, and representations of biochemical systems, and intend to improve the communication and sharing of M&S components.”

**Comment 9.** This is where I start to get confused on the rubric. I feel like the argument you are making here is that the information used to determine credibility needs to be widely available and easily understood. But why would this be so? If someone performed a credibility assessment of a model, why would the information they used to perform that assessment need to be widely available and easily understood?

For example, if I just wanted to know if the model was credible, I could just read their conclusion "The model is credible" or "The model is not credible". I don't need the VVUQ information to be widely accessible and easily understood for that. However, if the goal is so that others can perform their own credibility assessment, then I would certainly need that information and to be able to understand that information.

I thought that was the direction you were going with the rubric. The rubric was not there to evaluate the credibility of a model, but to evaluate whether or not that credibility could be easily evaluated by others (medical experts who are M&S experts, medical experts who are not M&S experts, non-medical experts, etc).

To me, this would make sense given the problem of lack of trust. Thus, instead of focusing on determining if a model could be trusted, this rubric recognized that that credibility decision should not be centralized to one organization, but would need to be made by multiple individuals at multiple organizations, and the rubric was designed to evaluate if the determination of the ten rules could be performed by various individuals, or if the determination of the ten rules could not be performed.

*In reference to “As Tatka et al. (6) noted in their review, standards for model annotation must become more widely accepted such that interoperability, reusability, comparability, and comprehension can be improved. Credible practice will also be enhanced when the information needed for simulation and parameter estimation is explicitly defined and stated. Lastly, reproducibility would not be possible without efficient dissemination of all artifacts and proper documentation on an open-source repository platform.” beginning on Line 76.*

**Response:** We thank the reviewer for their commentary. The point we are trying to convey in this section is that when an M&S study conforms to credible modeling practice guidelines at a high level, there is outreach to a wide range of stakeholders, thus with proper dissemination of documentation, one will largely be able to independently reproduce the M&S results.

**Action taken:** We have added additional text in the revised manuscript to clarify this point.

**Comment 9.** Is this referring to the authors of the Tatka paper, or the authors of this paper I am reviewing? Please make this clear.

*In reference to “The authors note” on Line 81.*

**Action taken:** We have clarified this sentence in the revised version of the manuscript to read “The authors of Tatka et al. (6) note”.

**Comment 10.** I very much agree, but the challenge is, as you have identified, you are using an ordinal scale, and I strongly believe that an ordinal scale only provides qualitative, not quantitative information.

Consider other ordinal scales. For example, movie posters use ordinal scale (e.g., four stars, two thumbs up) and restaurant ratings use ordinal scales (e.g., 2 Michelin stars). I do not believe those values can be considered to be quantitative because they are not tied to a the value of a quantity, only a general statement on the amount of that quantity. For example, consider the quantity of "goodness of a movie". If we compare a 4 star movie to a 2 star movie, we do not believe that the 4 star movie has twice the "goodness" of a 2 star movie. We think a 4 star movie has more "goodness" (i.e., it has a larger amount of goodness), but we can not put a quantity on that amount. Just that it is more.

Hence, any comparisons made with ordinal data must be qualitative in nature.

*In reference to “there is a lack of consensus on quantitative credibility scoring” on Line 82.*

**Response:** This is a fair point and we agree about the qualitative comparisons enabled by ordinal scoring. Your example and point about lack of quantitative/ratiometric meaning to the score is well taken. We kindly refer the reviewer to our response to Comment 3, in which we explain our intended use of an ordinal scale that is to be used with the rubric.

**Action taken:** We have clarified this point in the revised version of the manuscript.

**Comment 11.** To me, this is one of the most challenging parts of credibility assessment. It wasn't solved by the people who developed and evolved PCMM, it wasn't solved by NASA, I don't think its solved here, and I am personally convinced it can't be done because I don't think its mathematically possible to develop a metric on ordinal data. There are ways to develop things that look like metrics, but this method is not proposing that.

However, that was why I somewhat liked where I thought the rubric was going. Instead of trying to come up with a measure for credibility, come up with a measure to help you determine if the credibility can be determined by a wide audience.

*In reference to “While every effort was made to thoroughly describe and define the rules, it lacks a quantitative, rigorous, and repeatable metric.” beginning on Line 87.*

**Response:** We thank the reviewer for their comments and agree with their point. We would like to clarify that this section describes the need for a complementary rubric in addition to the rubric that we have outlined in this manuscript that could be used to address the credibility of a model as it pertains to validity, level of detail, and “correctness”. As the reviewer has pointed out, the development of such as rubric is very challenging. Therefore, the rubric we have proposed in this manuscript seeks to assess a modeling study’s conformance to M&S credible practices as it pertains to outreach capability.

**Comment 11.** The citation in the reference section seems off. Is this the correct way to reference V&V 40?

*In reference to citation (10).*

**Response:** We apologize for the oversight and agree with the reviewer that the V&V40 was incorrectly referenced. We have updated the reference in the revised version of the manuscript.

**Comment 12.** This reasoning seems very flawed. You are arguing that a rule-specific assessment is needed for each rule, but that such an assessment would be overly complex and would lead to inconsistent evaluation of the 10 rules.

So, you are not going to use a rule-specific assessment, even though it is likely needed? I feel like you are really arguing that the 10 rules are not good.

In other words, you are saying "To evaluate the Ten Rules correctly, we should evaluate each one individually. However, if we evaluate each rule individually, then our results won't make sense. Therefore, to make our results make sense, we will not evaluate the Ten Rules correctly."

*In reference to “become overly complex and unwieldy for consistently evaluating conformance to the Ten Rules.” beginning on Line 106.*

**Response:** We thank the reviewer for their comment. Here, we are still referring to the complementary rubric that would be used for assessing model credibility (validity, level of detail, and overall “correctness”) rather than the rubric we have outlined in this manuscript, which is to be used for assessing a modeling study’s conformance to M&S credible practices as it pertains to outreach capability.

**Action Taken:** We adjusted the relevant sentence in the text to clarify the issue.

**Comment 13.** This is very confusing.

Are you talking about communicating the results of the M&S? Or the results of the credibility assessment as provided by the answers to the Ten Rules.

I can't tell, and that information is extremely important to what the rubric is actually doing.

*In reference to “outreach of the results” on Line 110.*

**Response:** We agree with the reviewer that this verbiage is confusing. Thus, we have updated the text in the revised version of the manuscript to read “To address this issue, the Committee formulated a rubric based on the ability of the M&S to facilitate outreach to a wide range of stakeholders from context-specific M&S practitioners to policymakers.”

**Comment 14.** This term does not appear in the rubric.

*In reference to the term “policymakers” on Line 111.*

**Response:** We included the term “policy makers” in this sentence as to explain the wide range of stakeholders that should be considered when assessing outreach capability of a modeling study.

**Comment 15.** I have never seen them before, but to me the Ten Rules are a process used to ensure that the M&S developed is credible. If It did help disseminate that information, I think you wouldn't need this rubric.

*In reference to “The development of the Ten Rules facilitates such communication and understanding of computational modeling implementation and simulation results between stakeholders.” beginning on Line 112.*

**Response:** We appreciate the reviewer’s comment and have updated the text in the revised manuscript to clarify this point. A rubric is needed to assess how well a modeling study follows the Ten Rules. The sentence now reads “The development of the Ten Rules rubric facilitates such communication and understanding of computational modeling implementation and simulation results between stakeholders.”

**Comment 16.** This makes me think that the rubric is evaluating the communication of the M&S results and not the results of the credibility assessment performed by answering the Ten Rules.

*In reference to “the need for direct and clear communication of M&S results is essential.” on Line 112.*

**Response:** We kindly refer the reviewer to our responses to the previous comments, where we explain our intended use of the rubric.

**Comment 17.** I feel like this paragraph has gotten way off course. The first two sentences were on the rubric. The next two sentences had nothing to do with the rubric and are only about the Ten Rules. However, there is no link between the first two and last two sentences.

*In reference to the paragraph beginning on Line 109.*

**Response:** We thank the reviewer for their comment. After adjusting the text in the revised manuscript per Comment 15, the paragraph now clearly explains why the Ten Rules rubric was developed.

**Comment 18.** Are you arguing that each stakeholder be given the same credibility assessment framework (e.g., the Ten Rules) and the same VVUQ information such that each stakeholder can perform their own credibility assessment? Or are you arguing that one group should perform the credibility assessment and just disseminate that assessment to all stakeholders?

*In reference to “Most recently, the global response to the COVID-19 pandemic highlights the need for a systematic assessment of credible practice of M&S across this entire spectrum of stakeholders.” beginning on Line 119.*

**Response:** We thank the reviewer for their comment. In this sentence, we make a point that during the COVID-19 pandemic, the M&S community could have benefited from a more systematic method of assessing conformance to credible modeling practices. Having a credible practice assessment of the kind we developed here could have assisted a wider range of stakeholders in better understanding the context of use and limitations of these modeling studies.

**Comment 19.** Do you mean applying the rubric? Or do you mean that we are going to assess the rubric you developed?

*In reference to “assessing the rubric” on Line 143.*

**Response:** We thank the reviewer for their comment and have adjusted the text in the revised version of the manuscript to read “The remainder of the manuscript is organized as follows: First, we describe our process for developing and utilizing the rubric.”

**Comment 20.** So, the Ten Rules are the credibility framework an the rubric helps you determine if you have satisfied those Ten Rules?

*In reference to “in assessing the conformance to the Ten Rules” on Line 145.*

**Response:** We kindly refer the reviewer to our responses to the previous comments, where we explain our intended use of the rubric.

**Comment 21.** I don't understand how the rubric assesses the extent of conformance to the Ten Rules.

I agree it does provide information on if that information can be communicated, but I do not understand how you can claim it assess that information.

*In reference to “assesses” on Line 152.*

**Response:** We updated the text in the revised version of the manuscript as follows: “Our proposed rubric can be used to assess and communicate the extent of conformance to the Ten Rules for Credible Practice of M&S based on the capability of outreach to the biomedical and healthcare community (Table 2).”

**Comment 22.** This is where I am very confused. First, I can’t tell if the rubric has one dimension or three dimensions. That is, is Outreach Capability, Conformance Level, and Level Description three separate dimensions? For example, for the same simulation, and considering the same Rule being evaluated (here we will say Rule 5 – Version control) could I have a score of 0 for Outreach Capability, 4 for Conformance level, and 2 for Level Description? Or do I only have a single score?

And, if I have the single score, what does that score represent? For example, suppose I have perfect version control for a model/simulation. That is, I fully follow rule 5. However, now consider two different situations, the first in which I hide the fact that I perfectly follow rule 5 and the second in which I tell everyone I follow rule 5. Is this rubric meant to distinguish between those situations or not?

Conversely, considering the scenario in which I follow none of Ten Rules. However, I make it very clear that I do not follow any of the Ten Rules. Do I get rated high in the rubric because what I am doing can be understand by non-M&S practitioners? Or do I get low scores because I am not following the 10 rules?

Is the rubric supposed to evaluate if I have followed the Ten Rules, or if I have provided information such that someone else could evaluate if I have followed the Ten Rules? I do not understand.

*In reference to “Table 2. rubric for assessing conformance to the Ten Rules”.*

**Response:** We thank the reviewer for their comments. Outreach Capability, Conformance Level, and Level Description are not three separate dimensions but rather all related to a single interconnected concept of a modeling study’s conformance to M&S credible practice (Ten Rules). Each of the Ten Rules will be evaluated according to the rubric and given a single score. For instance, consider our assessment of Rule 5 – use version control. If the Outreach Capability is Comprehensive (score = 4), then the use of version control was implemented at the highest level with non-M&S practitioners familiar with the application domain and the indented context of use capable of understanding.

We now consider the two scenarios that you posed: (1) you have perfect version control for a model/simulation but you hide the fact that you perfectly follow Rule 5, and (2) you have perfect version control for a model/simulation but tell everyone that you follow Rule 5. In scenario 1, you would receive a conformance level score of 0 (Insufficient), as the information needed to assess conformance to the rule is missing (see Table 2). In scenario 2, you would receive a conformance level score of 4 (Comprehensive), as you have implemented a system to trace the time history of modeling and simulation activities including delineation of each contributors’ efforts to the highest degree such that it can be understood by non-M&S practitioners familiar with the application domain and the indented context of use.

If we then consider the scenario in which you follow none of the Ten Rules even if you make it very clear that you did not follow any of the Ten Rules, then you will receive a conformance level of 0 (Insufficient) because you have not followed any of the Ten Rules. By definition, lack of available information to assess conformance to any given rule will yield a score of 0 for that rule. In summary, the purpose of the rubric is to evaluate and assess an M&S study’s conformance to credible practice outlined in the Ten Rules, and the level at which the study communicates the conformance is based on outreach capability.

**Comment 23.** I don't know Outreach Capability is even in the rubric, you never talk about it. You seems to only use the Conformance Level... and I can't tell if Conformance Level 4 is Outreach Capability Level 4 or not.

*In reference to Outreach Capability in Table 2.*

**Response:** We kindly ask the reviewer to refer to our response to Comment 22.

**Comment 24.** What do (A), (B), and (C) correspond to in the figure? You have these letters here as if they mean something, but they are not associated with anything.

*In reference to Figure 1 caption “(A) The range of stakeholders that may utilize the rubric. (B) Example distribution of the stakeholders in the stakeholder assessment chart. (C) The conformance levels to each of the Ten Rules based on the extent of outreach to stakeholders along the M&S expertise and domain familiarity axes.”*

**Response:** (A), (B), and (C) correspond to the individual components within Figure 1. We have ensured that this can be seen in the revised version of the manuscript.

**Comment 25.** I very much disagree with what you are doing here. The numbers in the Conformance level are not quantities, but representations of a quality. They are still are ordinal set like "good, better, best". However, by using numbers, I feel like you may have tricked yourself into thinking that the numbers have added a quantitative aspect to the conformance level, and they cannot do that. If you are seeing "meaning" in the numbers, then that "meaning" in those numbers, that "meaning" would seem to be entirely fictitious because you should be able to use any ordinal set to represent those levels (e.g., A-F).

*In reference to “Therefore, a second development of the Ten Rules rubric was implemented. In this development, a scoring system was included such that for each rule, the level of conformance is given a numerical score” beginning on Line 179.*

**Response:** We thank the reviewer for their comment and kindly request the reviewer to reference our responses to the previous comments, where we explain our use of an ordinal scale to supplement the Ten Rules rubric.

**Comment 26.** I do not understand this at all. M&S credible practice conformance is based on the M&S itself and how it was created. M&S experience and scientific domain expertise is entirely independent of the M&S and dependent only on some external audience looking at the results.

*In reference to “but rather analyzes M&S credible practice conformance based largely on two dimensions: M&S experience and scientific domain expertise” beginning on Line 253.*

**Response:** We thank the reviewer for their comment. The purpose of the rubric is to assess a modeling study’s conformance to credible practices of M&S. The axes for which we consider a modeling study’s conformance are based on M&S experience and scientific domain expertise. A conformance score is thus determined based on outreach capability to the wide range of stakeholders given these two critical axes.

**Comment 27.** Is it a communication tool or an analysis tool? I can't tell which one it is. I don't see how it helps in the analysis of the ten rules, but I am not entirely sure how it communicates that information either.

*In reference to “We note that the rubric for conformance to the Ten Simples Rules is not an M&S practice accreditation process, but rather a communication tool for analyzing the robustness of the M&S practice employed” beginning on Line 254.*

**Response:** We kindly refer the reviewer to the previous comments in which we address the purpose of the rubric.

**Comment 28.** Okay, so Rule 2 is about using contextually appropriate data.

So lets pretend that I did not do that. I used data that was entirely inappropriate. But also assume that I made it clear the data was entirely inappropriate. I didn't hide anything, I was upfront with everything and explained it well. According to the rubric, I would get the highest "Conformance" score because I fully satisfy 1) the data being traceable, and 2) the experts can easily understand how the data was used. All of those experts would clearly see that I did not follow Rule 2, but actually following the Rule has no impact on the "Conformance Score" of the rule?

I am really confused.

*In reference to “M&S practice conforms to this rule at the highest level (Comprehensive) if:” beginning on Line 280.*

**Response:** We appreciate the reviewer for catching this flaw in our example, which resulted from our assumption that it would be obvious to first confirm whether the rule was being applied in accordance with its definition. But in brief, if you consider a scenario in which you used data that was entirely inappropriate but was very clear about the misuse of the data, you would still receive a score of 0 for Rule 2 – Use contextually appropriate data. This is because the rubric is to be used directly with the Ten Rules, which state for Rule 2 that one must employ relevant and traceable information in the development or operation of a model or simulation. Therefore, since you are insufficiently conforming to Rule 2 of the Ten Rules, you would receive a score of 0.

Action Taken: To address this concern, we have revised the manuscript by adding a general guideline outlining a systematic process for assessing conformance of the M&S practice to each rule. Specifically, we introduced three key questions that guide the evaluation of the M&S practice:

1. Whether the M&S practice employs the rule as defined by Erdemir et al. (2020),
2. The outreach capability of the rule’s implementation (i.e., which stakeholders can effectively engage with it), and
3. How easily different stakeholders can understand the extent to which the rule was applied.

Additionally, we clarified that if the answer to the first question is “no” or “uncertain,” the conformance score of the M&S practice for that rule must be assigned as 0 (Insufficient), ensuring consistency in evaluation. This new content is integrated into the section on “Assessment criteria for each rule” to enhance clarity and provide a structured workflow for applying the rubric.

We have also updated the Rule 2 example to conform to this guideline.

We believe this revision directly addresses the reviewer’s concern by making the rubric’s application process more explicit and actionable. Thank you for the valuable feedback.

**Comment 29.** I won't go into more details, but I think that representing the scores as numbers is fine. But then using the mathematical property of those numbers, which the scores clearly do not have, is going to lead to confusion. As it pretty much does every someone assumes that number from an ordinal system can be used in a ratio system.

*In reference to “Numerical scoring used in the rubric” section.*

**Response:** We thank the reviewer for their comment and kindly refer them to the previous comments in which we address the use of an ordinal scale to complement the rubric.

**Comment 30.** I have to stop my review here.

I did read the paper fully, but at this point, I do not understand what the rubric is trying to communicate, how it works, what it means, etc that I feel like my comments will become more repetitive than they already are.

**Response:** We thank the reviewer for their extensive commentary and have responded to all of the concerns raised by the reviewer as detailed above.

**Reviewer #2:**

The manuscript “A rubric for assessing conformance to the Ten Rules for credible practice of modeling and simulation in healthcare” by Vadigepalli et al describes a process for assessing the outreach capability of modeling and simulation documentation based on each of the previously established Ten Rules. In brief, outreach is scored from level 0 (“Insufficient”) to level 4 (“Comprehensive”). An aggregate score is also calculated by summing the scores, resulting in an overall score between 0 and 40. The rubric is demonstrated using COVID-19 modeling case studies from literature. Although assessment of individual Rules based on outreach capability differed among recruited review participants, aggregate scores mostly agreed within a few points, supporting the robustness of the rubric for assessing outreach (e.g., Table 7).

That said, I have significant concerns regarding language used to describe the rubric and potential for miscommunication and misinterpretation when used in practice.

**Comment 1.** Ten Rules Conformance versus Ten Rules Outreach Potential:

The purpose of the rubric requires explicit clarification. Two concepts are presented throughout the manuscript: 1) *technical* conformance to the Ten Rules, and 2) *outreach* potential of modeling study documentation based on each of the Ten Rules. From this reviewer’s perspective, the two concepts are orthogonal and should not be presented together. For example, a model with excellent predictive accuracy that conforms to the Ten Rules may not have supporting plain-English documentation, limiting outreach. Conversely, a terrible model with limited to no predictive accuracy could have very clear documentation that facilitates outreach. Other permutations are likewise possible (high conformance and outreach, low conformance and outreach).

However, several times throughout the manuscript, the concepts of Ten Rules conformance and outreach potential are intermingled. Line 109 of the Introduction explains the purpose of the proposed rubric:

“To address this issue, the Committee formulated a rubric based on the *ability of the M&S to facilitate outreach of the results to a wide range of stakeholders* from context-specific M&S practitioners to policymakers.” (emphasis added)

Elsewhere, the text implies the rubric assesses *conformance* to the Ten Rules. The title itself states: “A rubric for assessing *conformance* to the Ten Rules for credible practice…”.

The disconnect is perhaps more clearly demonstrated on lines 441-445:

“Rule #5 (Use version control): The model’s conformance was considered to be Extensive (score = 3) as the evolution of the model and the various versions are explicitly documented on GitHub. GitHub is a platform familiar to M&S practitioners but not necessarily to experts in the liver physiology and pathology domain. Hence, the conformance level was not considered Comprehensive (score = 4).”

Here, the score is lowered *not* because of lack of conformance to the Ten Rules’ Rule #5 for version control, but because of the perceived difficulty in communication and outreach without an accompanying explanation of git. Had the documentation included lay explanations of version control, but version control was poorly executed, could the highest outreach score of “Comprehensive” (5) have been achieved? This is potentially problematic. Additional excerpts that may be similarly confusing to readers are provided below.

As presented, the outreach rubric could lead decision makers interpreting scores to believe an inaccurate model is highly credible solely because the outreach capability score is high. This is potentially dangerous if decision makers are not provided clear context of the rubric and its purpose.

To resolve the concern, the reviewer recommends revising the manuscript to consistently present the scope of the rubric as “A rubric for assessing *outreach capability*” or similar and omitting or isolating mentions of technical conformance to the Ten Rules and model credibility. The scope of the outreach rubric should also be emphasized throughout the manuscript to avoid confusion for readers, including in the manuscript title.

**Response:** We thank the reviewer for their extensive commentary. We would like to point out that the purpose of the rubric is not to evaluate a model’s performance/credibility/”correctness” but rather to assess an M&S ***study’s*** conformance to credible practices outlined in the Ten Rules as it pertains to outreach capability. However, one must abide to the Ten Rules at the highest conformance level to be considered Comprehensive.

With regard to the scenario highlighted above where we address the conformance score of the liver physiology model to Rule #5 (Use version control), a score of “Comprehensive” was not attained because a proper system was not set up to trace the time history of M&S activities including delineation of each contributors efforts that could be understood by non-M&S practitioners familiar with the application domain and the intended context of use. Instead, the modeling study only received an “Extensive” score as the outreach capability was limited to M&S practitioners who may not be application-domain experts.

**Action taken:** We have updated the text in the revised version of the manuscript to clarify and explain the purpose of the rubric more explicitly. Additionally, we have adjusted the text in the revised version of the manuscript such that outreach capability and conformance to credible modeling practices are not to be confused with technical performance of a model.

**Comment 3.** Introduction Length: The Introduction as drafted is long (eight paragraphs, 5 pages). The manuscript would benefit from a more concise Introduction to summarize relevant background and motivate the need for the outreach rubric specifically.

**Response:** We thank the reviewer for their comment. In order to provide the reader with a comprehensive understanding of the rubric’s origin and purpose, we believe that the detailed explanatory aspects of the Introduction section can be very beneficial.

**Comment 4.** Additional excerpts related to rubric scope concern:

Line 34: “Here, we present a pragmatic rubric for assessing the conformance of an M&S activity to the TR”: Per the above, this does not appear to be true. Revise to clarify the scope of the rubric is limited to *outreach capability* (or similar wording).

Line 38: “We used the rubric to evaluate the *conformance* of two computational modeling activities” -> “We used the rubric to evaluate the *outreach capability* of two computational modeling activities”

Line 46: “Our findings demonstrate that the TR rubric represents a systematic tool for assessing the *conformance* of an M&S activity” -> “…assessing the *outreach capability* of…”

Line 233: “The rubric assesses the conformance to each of the Ten Rules based on the extent of outreach to each group”: Can a model conform to the Ten Rules but be poorly presented for outreach? Recommend rephrasing to omit mention of conformance.

See also Lines 137, 145, 152, Table 2, Figure 1 caption, 174, etc (135 total uses of the word “conformance”).

**Response:** We thank the reviewer for their comments. However, we believe that the purpose of the statements still holds true. The purpose of the rubric is to assess the conformance of an M&S activity to credible modeling practices i.e., the Ten Rules. The conformance ***level*** of a modeling activity to each of the Ten Rules is intrinsically dependent on the extent of outreach to stakeholders along the M&S expertise and domain familiarity axes.

**Action Taken:** Multiple edits have been made to the manuscript text in response to the above comments and Reviewer 1 comments to clarify this point.
